# Anti-Quorum Sensing Potential of Crude *Kigelia africana* Fruit Extracts

**DOI:** 10.3390/s130302802

**Published:** 2013-02-27

**Authors:** Hafizah Y. Chenia

**Affiliations:** School of Life Sciences, University of KwaZulu-Natal, Westville Campus, Private Bag X54001, Durban, 4001 KwaZulu-Natal, South Africa; E-Mail: cheniah@ukzn.ac.za; Tel.: +27-31-260-7796; Fax: +27-31-260-7809

**Keywords:** *Kigelia africana*, phytochemical extracts, quorum sensing inhibition

## Abstract

The increasing incidence of multidrug-resistant pathogens has stimulated the search for novel anti-virulence compounds. Although many phytochemicals show promising antimicrobial activity, their power lies in their anti-virulence properties. Thus the quorum sensing (QS) inhibitory activity of four crude *Kigelia africana* fruit extracts was assessed qualitatively and quantitatively using the *Chromobacterium violaceum* and *Agrobacterium tumefaciens* biosensor systems. Inhibition of QS-controlled violacein production in *C. violaceum* was assayed using the qualitative agar diffusion assay as well as by quantifying violacein inhibition using *K. africana* extracts ranging from 0.31–8.2 mg/mL. Qualitative modulation of QS activity was investigated using the agar diffusion double ring assay. All four extracts showed varying levels of anti-QS activity with zones of violacein inhibition ranging from 9–10 mm. The effect on violacein inhibition was significant in the following order: hexane > dichloromethane > ethyl acetate > methanol. Inhibition was concentration-dependent, with the ≥90% inhibition being obtained with ≥1.3 mg/mL of the hexane extract. Both LuxI and LuxR activity were affected by crude extracts suggesting that the phytochemicals target both QS signal and receptor. *K. africana* extracts with their anti-QS activity, have the potential to be novel therapeutic agents, which might be important in reducing virulence and pathogenicity of drug-resistant bacteria *in vivo*.

## 1. Introduction

Since plants live in environments with high microbial loads, it is not surprising that they have developed innovative protective mechanisms against bacterial infections [1]. Plant survival involves the production and release of secondary metabolites into their immediate environment, not only as a means of defense against potential pathogens but also as an offense against competing species. They are geared towards the production of antimicrobial compounds that limit the ability of microbes to produce the factors required for virulence and successful colonization [2]. Medicinal plants offer an attractive repertoire of phytochemicals with novel microbial disease-controlling potential, due to the spectrum of secondary metabolites present in extracts, which include phenolics, quinones, flavonoids, alkaloids, terpernoids and polyacetylenes [3].

Many phytochemicals are not highly effective as antimicrobial agents, instead they possess anti-pathogenic or anti-virulence properties, which are neither bactericidal nor bacteriostatic and therefore do not cause the development of resistant bacteria. Instead these compounds attenuate the expression of genes responsible for pathogenesis and virulence by interfering with quorum sensing (QS) and other related properties [3]. Quorum sensing is a communication system that allows bacteria to monitor their population density through the production and sensing of small signal molecules called autoinducers. In Gram-negative bacteria, autoinducer molecules include acyl-homoserine lactones (AHLs) which are synthesized by the members of autoinducer synthases (LuxI homologues). The synthesized signaling molecules are secreted out of the cell and bind with specific receptor proteins (LuxR homologues) of neighboring bacterial cell walls [4]. QS systems regulate a wide spectrum of cellular and physiological processes including bioluminescence, adhesion and biofilm formation, antibiotic production, virulence factor expression, pigment production, motility, exopolysaccharide production, formation and activity of many degradative enzymes in animal, fish and plant pathogens [1]. Given that QS is an important process in bacterial survival, pathogenicity and virulence, the development of therapeutic drugs which prevent or manage bacterial pathogenesis by inhibiting bacterial QS is critical.

QS can be targeted in a number of ways, including inhibition of AHL molecule biosynthesis, degradation of AHL molecules by bacterial lactonases and acylases, and/or using small molecules to block the activation of AHL receptor protein [4,5]. The ideal QS inhibitors have been defined as chemically stable and highly effective low-molecular-mass molecules, which exhibit a high degree of specificity for the QS regulator without toxic side effects on either the bacteria or an eventual eukaryotic host [4]. Both synthetic and natural compounds are able to disrupt QS-regulated behaviors of bacteria. The first group includes methylthioadenosine analogs, whereas halogenated furanones, dietary phytochemicals from fruits, herbs, and spices such as cinnamaldehyde, garlic and vanilla extracts and phytochemicals from medicinal plant extracts, are among the natural ones [1,4,5]. Unfortunately, the halogenated furanones are too reactive and may be too toxic for treatment of bacterial infections in humans [6] and have been also shown to be lethal to rainbow trout. There is thus an increasing need for the identification of novel non-toxic QS inhibitors, which could result in the development of novel non-antimicrobial drugs for treating bacterial diseases in humans, in agriculture, aquaculture and animal husbandry.

Quorum sensing inhibitory (QSI) compounds have been identified from a wide range of natural resources, particularly medicinal plants, edible herbs, fruit and vegetables [1,3,6,7], as well as spices [1,8]. Natural products are promising sources of QSI compounds that can potentially inhibit QS regulation of bacterial colonization and virulence factor production. It is also possible that some of the antimicrobial properties of phytochemicals may be attributed to QS inhibition, which may not be related to growth inhibition of the microorganism [1]. Such anti-pathogenic compounds, in contrast to antimicrobial agents, are neither bactericidal nor bacteriostatic and reduce the risk of resistance development [7,9]. Compared with conventional antimicrobial agents, QS inhibitory compounds that do not kill or inhibit microbial growth are less likely to impose a selective pressure for the development of resistant bacteria. Phytochemicals often have multiple therapeutic effects and one of their mechanisms of action might be QS inhibition or modulation, whereby they attenuate the expression of virulence genes responsible for pathogenesis and the establishment of successful infections by interfering with bacterial communication systems [1,7]. This coupled with their extensive usage against infectious diseases both in traditional and modern medicines [3] makes them attractive in drug discovery

**Figure 1.**
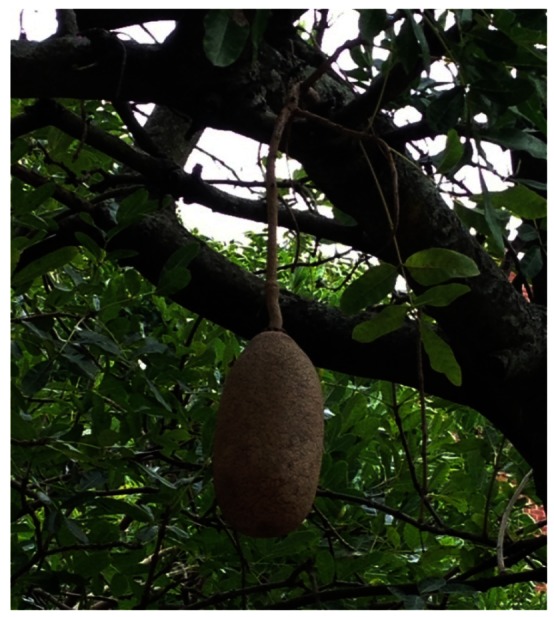
*Kigelia africana* (Lam.) Benth., commonly known as the sausage tree, of the Bignoniaceae family with hanging fruit pod.

*Kigelia africana* (Lam.) Benth., of the Bignoniaceae family and commonly known as the sausage tree, is found in south, central and West Africa (Figure 1). This plant has a long history as a medicinal plant used by many rural and African countries. It is often used as a topical application on wounds and abscesses, for the treatment of skin ailments like acne, fungal infections, boils, psoriasis and eczema, and for the treatment of sexually transmitted diseases. Internally, it is used for treatment of dysentery, ringworm, tape-worm, post-partum haemorrhage, malaria, diabetes, pneumonia, and toothaches. Ripe or unripe *K. africana* fruits are dried and powdered and are used as dressing for wounds and ulcers, haemorrhoids, for rheumatism, as a purgative, to increase lactation in breast-feeding mothers and for skin-firming properties [10,11]. Various parts of the plant, including the fruit, are used either in a powder form or as aqueous or ethanolic infusions which are drank or applied to the affected body part [10]. The antimicrobial properties of *K. africana* leaves, fruits and stem-bark against Gram-negative and Gram-positive bacteria have been investigated [10], giving credence to the use of this plant in traditional medicine. The medicinal properties associated with *K. africana* are due to the presence of numerous secondary metabolites, including iridoids, flavonoids, naphthoquinones, meroterpenoid naphthoquinones, coumarin derivatives, lignans, sterols, flavonoids, furanones, furonaphthoquinones and volatile constituents [10,11]. Although the *K. africana* fruit extracts' antimicrobial properties are well-established, no information is available on its potential anti-QS activity. Thus the quorum sensing (QS) inhibitory activity of *K. africana* fruit extracts was investigated using the *Chromobacterium violaceum* and *Agrobacterium tumefaciens* biosensor systems.

## 2. Experimental Section

### 2.1. Maintenance of Bacterial Isolates

*Chromobacterium violaceum* and *Agrobacterium tumefaciens* biosensor system strains were used in this study. In the wild-type strain *Chromobacterium violaceum* ATCC 12472, production of a purple pigment, violacein, is under control of a QS system. This wild-type strain produces and responds to the cognate autoinducer molecules, *N*-hexanoyl-L-acylhomoserine lactone (C6-AHL) and C4-AHL, produced by the autoinducer synthase CviI. These AHLs bind to the receptor CviR and this complex triggers the expression of violacein production [12]. *C. violaceum* ATCC 12472 is used to detect potential quorum signal inhibitors. *C. violaceum* CV026 is a QS-bioassay organism that is unable to synthesize its own C6- AHL, but it retains the ability to respond to C4- and C6-AHLs. The CviR receptor of CV026 recognises C6-AHL as the cognate signal and is sensitive to short and medium-chain length AHLs [13]. *C. violaceum* ATCC 31532 is a C6-AHL over-producer and is used as a positive control for CV026.

The *Agrobacterium tumefaciens* biosensor system comprised of two *A. tumefaciens* strains A136 and KYC6. *A. tumefaciens* A136 (pCF218)(pCF372) is a bioassay strain for a range of AHLs, and was maintained on LB + spectinomycin (50 ug/mL) and tetracycline (4.5 ug/mL). It has a mutation in *traI* and consequently does not produce AHLs. The plasmid pCF218 contains *traR* expressed from the *tetR* vector promoter, and the plasmid pCF372 contains the *traI* promoter transcriptionally fused *to lacZ*. It overexpresses TraR, which binds exogenous AHLs and then activates the expression of the *traI-lacZ* gene fusion. The TraR receptor of *A. tumefaciens* detects a broad range of AHLs both oxo, hydroxyl, and unsubstituted. Strain KYC6 is a 3-oxo-C8- and 3-oxo-C6-AHL over-producer (null mutation in *traM*) and is used as a positive control for the AHL bioassay [14].

All strains were grown on Luria-Bertani (LB) agar, with or without antibiotics, at 30 °C, and were maintained on LB agar plates at room temperature for short-term storage and for long-term storage in LB broth containing glycerol at −70 °C.

### 2.2. Preparation of Crude Fruit Extracts

*Kigelia africana* fruits (Figure 1) were collected around the Westville Campus of University of KwaZulu-Natal. A voucher specimen of the *K. africana* plant (voucher specimen Chenia 1) is archived in the Ward Herbarium, University of KwaZulu-Natal, Westville Campus (International herbarium acronym UDW). Material was chopped, oven-dried at 60 °C, milled to yield a finely ground material and stored in polythene bags at 4 °C. Crude extracts were prepared by exhaustive sequential extraction with ethyl acetate, dichloromethane, hexane and methanol by maceration and continuous shaking on an orbital shaker at room temperature for 48 h [15]. These extraction solvents were chosen to most efficiently extract the diverse phytochemicals contained with the *K. africana* fruit material. Solvent extracts were concentrated using a vacuum rotary evaporator, dried, dissolved in dimethyl sulfoxide (DMSO) to a final concentration of 100 mg/mL and stored at 4 °C.

### 2.3. Antimicrobial Susceptibility Testing

Antimicrobial susceptibility to four crude *K. africana* fruit extracts was determined using the disc diffusion method. Blank discs (MAST, UK) were impregnated with 4 mg/mL (40 μL of 100 mg/mL stock) of crude *K. africana* ethyl acetate (EX 1), dichloromethane (EX 2), methanol (EX 3) and hexane (EX4) extracts and allowed to dry. Bacterial isolates were grown overnight on LB agar plates and the turbidity of cell suspensions were adjusted equivalent to that of a 0.5 McFarland standard. These were used to inoculate Mueller-Hinton (MH) agar plates by streaking swabs over the entire agar surface followed by the application of the respective phytochemical extract discs [16]. Plates were then incubated for 21 h at 30 °C. Testing was done in duplicate and tetracycline (TE30) and ampicillin (AMP10) discs were used as standard antimicrobial agent controls. Zone diameters were determined and averaged. The following zone diameter criteria were used to assign susceptibility or resistance to phytochemicals tested: Susceptible (S) ≥ 15 mm, Intermediate (I) = 11 – 14 mm, and Resistant (R) ≤ 10 mm. Criteria for assigning susceptibility or resistance to AMP10 was as follows: (S) ≥ 17 mm, (I) = 14–16 mm, (R) ≤ 13 mm, while those for TE30 were: (S) ≥ 19 mm, (I) 15–18 mm, (R) ≤ 14 mm [16].

### 2.4. Quorum Sensing Inhibition

#### 2.4.1. Qualitative Agar Diffusion Assay

The anti-QS potential of the four crude *K. africana* fruit extracts was detected using the wild-type pigmented biosensor strain *C. violaceum* ATCC 12472. Five milliliters of molten soft LB agar (0.3% w/v) was inoculated with 50 μL of *C. violaceum* ATCC 12472 grown overnight in Luria-Bertani (LB) broth. The agar-culture solution was immediately poured over the surface of pre-warmed LB agar plates [17]. Five, 20 and 40 μL (0.5, 2 and 4 mg/mL, respectively) of each 100 mg/mL extract was pipetted on sterile paper discs, dried and placed on the solidified agar. The plates were incubated overnight at 30 °C and examined for violacein pigment production. QS inhibition was detected by a colorless, opaque, but viable halo around the discs (loss of pigmentation). Purified halogenated furanone (10 and 20 μg/mL), cinnamaldehyde (50, 100 and 200 μg/mL) and vanillin (125, 250 and 500 μg/mL) were used as known QS inhibitors [5]. DMSO was used as a control.

#### 2.4.2. Quantitative Anti-QS Activity—Violacein Inhibition

Quantitative evaluation of QS inhibitory activity of the four crude *K. africana* extracts was carried out based on their ability to inhibit the production of purple pigment violacein by *C. violaceum* ATCC 12472 [13,17]. The strain was cultured aerobically in LB at 30 °C with or without the addition of increasing concentrations of the bioactive phytochemicals (EX 1–4: 0–8.2 mg/mL (0, 0.33, 0.66, 1.31, 1.97, 3.93, 6.56 and 8.2 mg/mL)). Cinnamaldehyde and vanillin (0–6.25 mg/mL; Sigma, St. Louis, MO, USA) were used as QSI-positive controls.

One mL of an overnight culture of *C. violaceum* ATCC 12472 was centrifuged (13,000 rpm, 10 min) to precipitate the insoluble violacein. The culture supernatant was discarded and the pellet was evenly resuspended in 1 mL of DMSO [9]. The solution was centrifuged (13,000 rpm, 10 min) to remove the cells and the violacein was quantified at OD_585nm_ using a UV**-**Vis spectrophotometer (UV-1800, Shimadzu, Kyoto, Japan). The percentage of violacein inhibition was calculated by following the formula: Percentage of violacein inhibition = (control OD_585 nm_ – test OD_585 nm_/control OD_585 nm_) × 100 [3].

#### 2.4.3. Qualitative Modulation of QS Activity

The effect of the four crude *K. africana* extracts EX1 – EX4 (2 mg/mL; 20 μL of 100 mg/mL stock) on modulation of AHL activity and inhibition of AHL synthesis was determined using an agar diffusion double ring assay [1] and the *A. tumefaciens* A136 biosensor system (strain A136 was the biosensor, while strain KYC6 was the over-producer). A modification of the assay involved the use of *C. violaceum* ATCC 12472 as AHL over-producer for the *A. tumefaciens* A136 biosensor strain. Discs impregnated with cinnamaldehyde (500 μg/mL), vanillin (200 μg/mL) and water were used as controls.

To observe the extent of QS activity in the A136 system, prior to inoculation with bacteria, 20 μL of 5-bromo-4-chloro-3-indolyl-β-D-galactopyranoside (X-gal, 20 mg/mL in DMSO) was spread evenly on LB agar plates and allowed to dry for 60 min. Sub-inhibitory concentrations (2 mg/mL) of the four extracts were placed on absorbent, sterile filter paper disks and the AHL over-producer and biosensor strains inoculated in concentric circles in proximity to the impregnated disks [1].

To test for potential LuxI inhibition, the AHL over-producer was placed in close proximity to the test substance and the AHL biosensor placed distally. To test for LuxR inhibition, the location of the AHL over-producer and biosensor strains was reversed. In either case, potential QSI activity results in a lower signal from the AHL biosensor than from the over-producer [1].

### 2.5. Statistical Analysis

All experiments were carried out in triplicate. The differences in violacein inhibition with and without the addition of varying concentrations of extracts was determined using pair-wise testing based on Student's *t*-tests using SigmaStat 3.5 (Systat Software Inc., San Jose, CA, USA), with *p* ≤ 0.05 being considered significant. The difference in violacein inhibition mean values between extracts was determined using One-way repeated measures ANOVA with *p* ≤ 0.05 being considered significant. To isolate the extract or extracts that differ from the others, the Holm-Sidak multiple pairwise comparison procedure was carried out, with *p* ≤ 0.05 being considered significant.

## 3. Results and Discussion

Bacterial intracellular communication, or QS, regulates the pathogenesis of many organisms important from a medical, agricultural and veterinary perspective. Common mechanisms of QS interference include inhibition of signal biosynthesis or inhibition of activity of AHL-producing enzymes, enzymatic signal degradation, and competitive/non-competitive inhibition of signal receptors [4,5]. Various species of marine algae and higher plants, including traditional medicinal plants and dietary fruits, herbs, and spices have been shown to interfere with bacterial QS and attenuate bacterial pathogenicity [3,6,7,9,18]. Given the use of *K. africana* fruit in traditional medicine and their antimicrobial effects [10], *K. africana* fruit extracts were screened for their anti-QS activity.

### 3.1. Antimicrobial Susceptibility Testing

**Figure 2.**
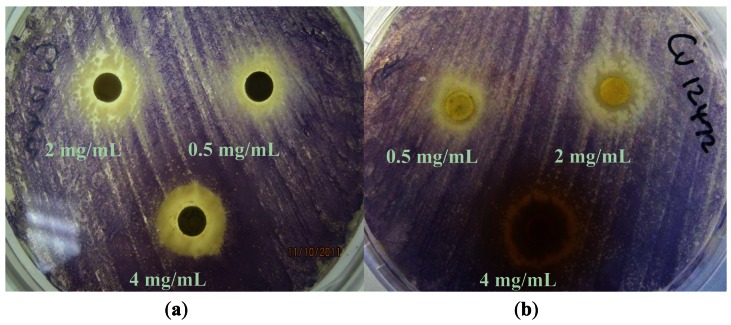
Antimicrobial activity of *Kigelia africana* ethyl acetate (**a**) and methanol extracts (**b**) [0.5, 2 and 4 mg/mL] against purple-pigmented *Chromobacterium violaceum* ATCC 12472. Translucent zones indicate inhibition of growth, while opaque zones represent inhibition of violacein production.

Based on their zone diameters, resistance was observed to all four extracts for *A. tumefaciens* strains A136 and KYC6 (Table 1). All *C. violaceum* strains demonstrated resistance to the ethyl acetate (EX 2) and dichloromethane (EX 2; Figure 2(A)) extracts, and intermediate susceptibility to the methanol (EX 3; Figure 2(B)) and hexane (EX 4) extracts (Table 1). Zones of opaque growth (indicative of QS inhibition) were observed for *C. violaceum* ATCC 12472 for all extracts in a concentration-dependent manner (Figure 2).

### 3.2. Quorum Sensing Inhibition

#### 3.2.1. Qualitative agar Diffusion Assay

**Figure 3.**
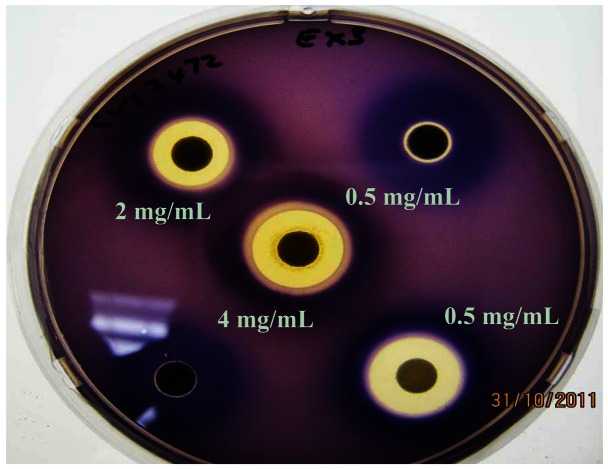
Agar diffusion bioassay of 0.5, 2 and 4 mg/mL *Kigelia africana* methanol extract (EX 3) using *Chromobacterium violaceum* ATCC 12742, depicting both anti-quorum sensing (non-pigmented zones) and antimicrobial activities (translucent zones). *K. africana* methanolic extract displayed different active principles, *i.e.*, antimicrobial activity indicated by inner clear ring and quorum sensing inhibition indicated by outer non-pigmented ring, in a concentration-dependent manner. Furanone and oxo-dodecanoyl AHL served as controls (bottom right-hand corner and left-hand corners, respectively).

*C. violaceum* synthesizes the violet pigment violacein as a result of QS. Loss of purple pigmentation of *C. violaceum* ATCC 12472 in the vicinity of the plant extracts was indicative of QS inhibition by the plant extracts (Figure 3). Where the extracts inhibited the growth of bacteria, clear inner zones of inhibition were observed (Figure 2). The outer colorless zone of inhibition observed was opaque and not transparent, indicating that the halos around the discs were caused by inhibition of QS, not inhibition of cell growth (Figure 3).

As expected, zones of QS inhibition were observed with the controls furanone, cinnamaldehyde and vanillin. All four *K. africana* extracts screened for anti-QS activity displayed violacein inhibition. The anti-QS activity was concentration-dependent (Figures 2 and 3), with an increase in the size of the colourless QS inhibition zones as concentration of extracts was increased.

#### 3.2.2. Quantitative Anti-QS Activity—Violacein Inhibition

**Figure 4.**
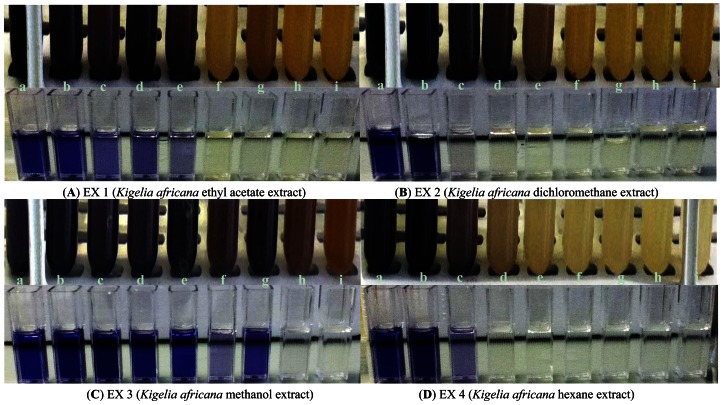
Concentration-dependent inhibitory effects of four *Kigelia africana* extracts, EX 1–EX 4, on violacein production by *Chromobacterium violaceum* ATCC 12472. (**A**–**D**): Effect of *K. africana* extracts EX 1–EX 4 on violacein production by *C. violaceum* ATCC 12472. (**a**) Untreated control; (**b**–**i**) extract-treated cultures showing progressive reduction in violacein production at the concentrations of 0–8.2 mg/mL, respectively.

**Figure 5.**
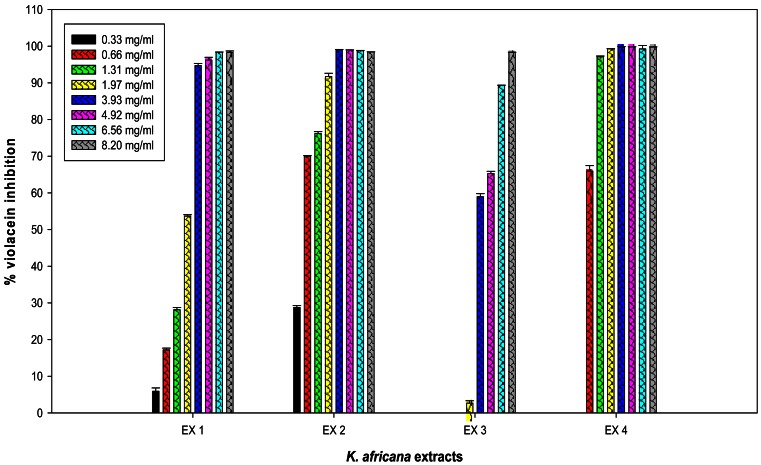
Quantitative analysis of the concentration-dependent inhibitory effects of four *Kigelia africana* extracts, EX 1–EX 4, on violacein production by *Chromobacterium violaceum* ATCC 12472. Cultures were grown in the presence of 0–8.2 mg/mL of respective *K. africana* extracts: EX 1—ethyl acetate extract, EX 2—dichloromethane extract, EX 3—methanol extract and EX 4—hexane extract. Data are the average of three triplicate independent experiments and SD are shown.

The inhibitory effect of all four crude extracts (0.33 mg/mL–8.2 mg/mL) on violacein pigment production was also measured spectrophotometrically and quantified (Figure 4). A concentration-dependent inhibition of violacein production by *C. violaceum* ATCC 12472 was observed with three *K. africana* crude extracts (EX 1, EX 2 and EX4; Figure 5), without inhibition of bacterial growth (Figure 4). A similar concentration-dependent inhibition of violacein production has been reported with methanolic extracts of dried *Capparis spinosa* fruit [8], *Cuminum cyminum* extract [3] and aqueous *Moringa oleifera* leaf and fruit extracts [6], without inhibiting bacterial growth.

The inhibitory effect on violacein production ranged from 5.9–98.28% for EX 1; 28.77–98.42% for EX 2; 2.69–98.3% for EX 3 and 65.92–99.76 for EX 4 (Figure 5). An 88% inhibition in violacein production was obtained with 2 mg/mL of the *C. spinosa* methanolic extract [8], while 2 mg/mL of the methanolic *C. cyminum* extract resulted in 90% inhibition [3].

In the present study, the four *K. africana* fruit extracts displayed varying levels of potency, with EX 4 being the most potent, demonstrating 97% inhibition of violacein production with 1.31 mg/mL (Figure 5), with an IC_50_ of 0.58 mg/mL. A gradual decrease in the production of violacein content was observed with increasing concentrations of *K. africana* EX 1 and EX 2 (Figure 5), with 90% inhibition of violacein production being observed with 1.97 mg/mL and 3.93 mg/mL, respectively. The IC_50_ for EX 1 and EX 2 was 1.89 mg/mL and 0.50 mg/mL, respectively. The methanolic extract (EX 3) was the least effective, producing ≥90% inhibition with 6.56 mg/mL of EX 3 (Figure 5), and an IC_50_ of 3.45 mg/mL. The quorum sensing inhibition effect was thus observed in the following order: EX 4 (hexane) > EX 2 (dichloromethane) > EX 1 (ethyl acetate) > EX 3 (methanol). With the exception of EX 3 at concentrations of 0.33–1.31 mg/mL, all violacein inhibition mean values at the varying concentrations of extracts tested were statistically significant (*p* ≤ 0.05). The differences in the mean values among the extracts was greater than would be expected by chance; there was a statistically significant difference (*p* ≤ 0.001). A statistically significant difference in means were observed between EX 2 and EX 3 (*p* = 0.009), as well as between EX 4 and EX 3 (*p* = 0.010).

The methanolic extract was more effective in its antimicrobial activity (at a concentration of 10 mg/mL) against various Gram-negative aquaculture pathogens of the genera *Aeromonas, Chryseobacterium, Flavobacterium*, and *Myroides* (unpublished data). The hexane (EX 4) and dichloromethane (EX 2) extracts were most effective in reducing violacein production (Figure 5), but had no or limited antimicrobial activity against aquaculture pathogens (unpublished data).

Adonizio *et al*. [7] and Koh and Tham [19] have observed that some medicinal plant compounds have both antimicrobial and anti-QS activity. This was also observed with the methanolic extract EX 3, which demonstrated both QS inhibitory activity as well as antimicrobial activity (Figures 2 and 3). The presence of numerous secondary metabolites, *i.e.*, naphthoquinones, a furanone derivative, a furonaphthoquinone, 11 iridoids, 3β,19α-dihydroxyurs-12-ene-28-oic acid, caffeic acid and chlorgeric acid, 6-*p*-coumaroyl-sucrose, together with a diverse group of 11 phenylpropanoid and phenylethanoid derivatives and a flavonoid glycoside may be associated with the medicinal properties attributed to *K. africana* fruit extracts [10,11]. The difference in the *K. africana* fruit extracts' efficacy as QS inhibitors might be explained by the extraction solvents which selectively affect the composition of active phytochemicals. Different solvents, like hexane (more non-polar) and methanol (more polar) were used in the extraction process due to their ability to extract different phytochemicals of varying polarity [20]. Grace *et al.*[21] observed that the antibacterial activity of *K. africana* fruits against Gram-positive and Gram-negative bacteria was due to the antimicrobial effects of a mixture of three fatty acids (palmitic acid, nonanoic acid and 8-heptadecenoic acid) in the ethyl acetate fruit extract. Dichloromethane-extracted *K. africana* fruit extracts predominantly contain two components, norviburtinal, and isopinnatal, which accounts in part for its cytotoxic effects, as well as β-sitosterol [10]. Higgins *et al*. [11] identified the compounds demethylkigelin and kigelin in a hexane-soluble fraction of the ethanol/H_2_O-extracted fruit material, with further fractionation revealing the presence of fatty acids, both saturated and unsaturated, in their free form and as glycerol esters, including oleic acid [unsaturated, CH_3_(CH_2_)_7_CH = CH(CH_2_)_7_COOH] and heneicosanoic acid [saturated, CH_3_(CH_2_)_19_COOH], as well as the phenolic compound, 3-hydroxy-4-methoxycinnamic acid and the furonaphthoquinone, 2-(1-hydroxyethyl)naphtho[2,3-b]furan-4,9-dione. The QSI effect is most likely due to the effect of the furanone derivative, 3-(20-hydroxyethyl)-5-(200-hydroxypropyl)-dihydrofuran-2(3H)-one [22] and the furonaphthoquinone [11], which have been isolated from *K. africana* methanolic fruit extracts. Like the brominated furanones, they could inhibit AHL-regulated behaviors by binding competitively to the AHL receptor protein. It is also possible that the anti-QS activity of these extracts may be the result of the multi-target action of the various phytochemical components of the fruit extracts. An example of this type of QSI activity is demonstrated by garlic extracts which contain at least three different QS inhibition compounds [4].

#### 3.2.3. Qualitative Modulation of QS Activity

In the AHL system, the signal-generating LuxI homolog, the *N*-acylhomoserine lactone molecule itself, and the signal receptor LuxR are potential targets. Many natural extracts inhibit QS by interfering with the AHL activity by competing with them due to their structural similarity and/or accelerating the degradation of the LuxR/LasR receptors of the AHL molecules [1]. A second level of modulation involves modulating the synthesis of AHL molecules by decreasing the expression of the LuxI family of synthases or the ability of phytochemicals to competitively or non-competitively inhibit LuxI activity. To determine whether potential inhibitors target AHL synthesis (via LuxI) or AHL response (via LuxR), a double ring bioassay was carried out using the *A. tumefaciens* biosensor system, at a sub-inhibitory concentration because of simultaneous antimicrobial action displayed by methanol extract EX 3.

β-galactosidase expression in *A. tumefaciens* A136 is under the control of QS and is expressed in response to the presence of AHL molecules secreted by the AHL over-producer KYC6. QS inhibitory compounds cinnamaldehyde, halogenated furanone, and vanillin, decreased the secretion of the β-galactosidase and blue pigment formation from the biosensor A136 for both LuxI and LuxR assays, in comparison to the control (water-impregnated discs) which did not contain extract. The low-levels of enzyme activity as well as decreased AHL synthesis were thus indicated by decreased X-gal hydrolysis and blue pigment formation.

**Figure 6.**
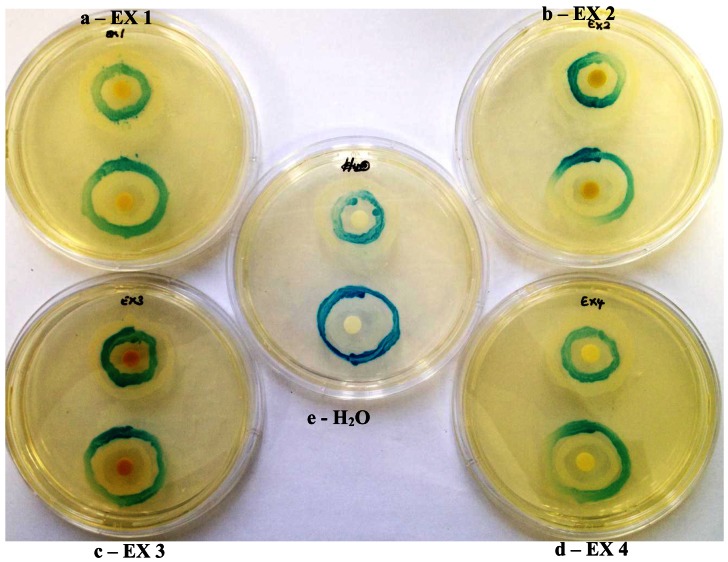
Quorum sensing inhibition by sub-inhibitory concentrations (2 mg/mL) of *Kigelia africana* extracts (EX 1–EX 4; a–d) by modulation of AHL receptor activity (LuxR; up) and AHL synthesis (LuxI; down) in the double ring agar diffusion assay with the *Agrobacterium tumefaciens* A136/KYC6 biosensor system. The control was a disc impregnated with 20 μl of sterile distilled water (E).

Similarly all four *K. africana* extracts, displayed varying levels of QS modulation (Figure 6), which was in keeping with the varying levels of violacein inhibition observed for each of the extracts at the sub-inhibitory concentration of 2 mg/mL (Figure 5). Extract EX 2 displayed both LuxI and LuxR modulation, although LuxI modulation was to a greater degree (Figure 6). Decreased secretion of β-galactosidase from the biosensor strain was observed, resulting in decreased X-gal hydrolysis and blue pigment formation. With EX 1, both LuxI and LuxR modulation was also observed but to a lesser degree, especially with the LuxR modulation.

The mechanism of these *K. africana* fruit extracts QSI activity thus appeared to be the additive effect of the ability of phytochemicals to interfere with the reception of AHL and to modulate the synthesis of AHLs. This has been observed previously with dietary phytochemicals [1], as well as with *Camellia sinesis* extracts [18]. The extracts probably act at different hierachial positions in the overall QS cascade, since they contain not a single phytochemical but potentially a group of QS-modulating compounds, allowing them to affect QS at multiple levels [18], as has been observed previously with garlic [4] and *C. sinsensis*[4] extracts.

Extracts EX 3 and EX 4 displayed only LuxI modulation (AHL synthesis), with EX 4 producing a greater modulation effect with the *A. tumefaciens* A136/KYC6 biosensor system (Figure 6). This was indicative of the respective extracts affecting 3-oxo-C8- and 3-oxo-C6-AHL synthesis only.

**Figure 7.**
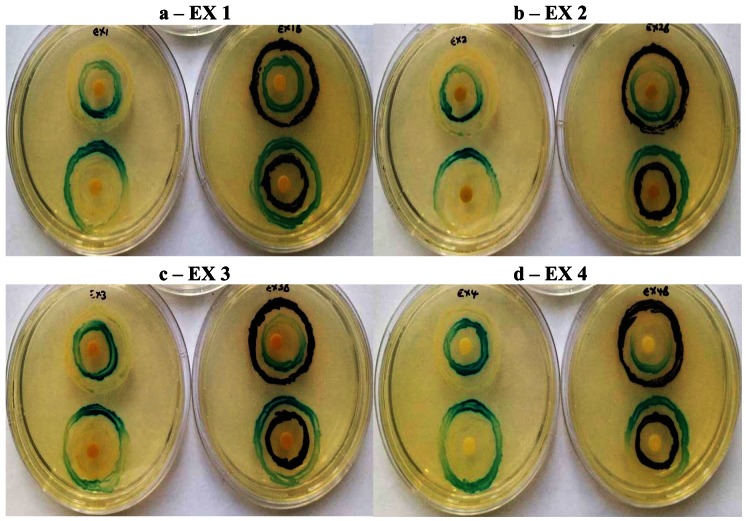
Quorum sensing inhibition by sub-inhibitory concentrations (2 mg/mL) of *Kigelia africana* extracts (EX 1–EX 4; a–d) demonstrating modulation of AHL receptor activity (LuxR; up) and AHL synthesis (LuxI; down) in the double ring agar diffusion assay with the *Agrobacterium tumefaciens* A136/KYC6 biosensor system (left) and *A. tumefaciens* A136/*Chromobacterium violaceum* ATCC 12472 combination (right).

Screening for QSI activity can also be improved by using multiple biosensor systems as the ability to detect QS inhibition depends on the reporter systems used [7,19]. The use of two biosensor systems eliminates QSI activity as a function of violacein production downstream of the QS system, while activity with *A. tumefaciens* alone suggests QSI effect on long chain signaling molecules and allows the identification of broad-spectrum QS inhibitors [7]. In the double ring assay where *C. violaceum* ATCC 12472 was used as the over-producer with the *A. tumefaciens* biosensor strain (Figure 6), both LuxI and LuxR modulation were obtained with extracts EX 4, EX 3 and EX 2 suggesting a stronger inhibitory effect on the synthesis and reception of short-chain AHLs (C4-AHL and C6-AHL). The *A. tumefaciens* A136 LuxR modulatory effect was greater when using *C. violaceum* ATCC 12742 in comparison to the *A. tumefaciens* over-producer strain KYC6 (Figure 7). Since all four extracts were effective at inhibiting QS mediated by two different AHL over-producers, it may be assumed that the responsible compounds have multiple or broad-spectrum effects, and are able to inhibit multiple bacterial QS systems which are mediated by diverse AHL molecules.

## 4. Conclusions

*K. africana* fruit extracts have a long history of use in African countries, for a wide range of medical complaints as well as for cosmetic purposes [10]. The present study is the first to identify the anti-QS activity of *K. africana* fruit extracts, which complements its documented antimicrobial, antineoplastic, and anti-inflammatory activities. Not only are these compounds non-toxic, but they are from a sustainable source with potential multiple applications [10]. The major advantage of using a *K. africana* fruit extract phytochemical-based anti-virulence therapy strategy is that it would circumvent the problem of resistance, associated with the use of conventional antimicrobial agents, since it specifically interferes with the expression of QS-associated virulence traits rather than being bactericidal. Inhibition of QS offers new hope in combatting multidrug-resistant bacteria, with potential application in many different fields, including aquaculture, medicine, agriculture, and food technology. Of importance is the ability of QS inhibitors to potentiate the effect of available antimicrobial agents and to inhibit biofilm formation, thus short-circuiting the infection process by evading the onset of bacterial pathogenesis. Future work should entail identifying the compounds in all four *K. africana* extracts to pinpoint the exact compound/s mediating the QS inhibitory effect, as well as assessing the mode of action and its anti-pathogenic efficacy in an experimental animal model. The furonaphthoquinones should be selectively isolated and examined for their anti-QS and anti-biofilm potentials. Alternatively it might be that the QSI effect and successful eradication of communicating pathogens involves the synergistic action of multiple bioactive phytochemicals in the *K. africana* fruit extracts.

## Figures and Tables

**Table 1.** Zones of inhibition (mm) obtained with 4 mg/mL of *Kigelia africana* extracts as well as standard antimicrobial agents, ampicillin and tetracycline. Response to tested compounds is indicated by S (sensitive), I (intermediate susceptibility) and R (resistant)

	**Zone diameters (mm) and associated susceptibility phenotypes**											
	EX 1 a		EX 2 a		EX 3 a		EX 4 a		AMP10 a		TE30 a	
***Chromobacterium violaceum*ATCC 12472**	8	**R**	8	**R**	13	**I**	12	**I**	0	**R**	29	**S**
***Chromobacterium violaceum*ATCC 31532**	8	**R**	7	**R**	14	**I**	7	**R**	0	**R**	22	**S**
***Chromobacterium violaceum*CV026**	10	**R**	8	**R**	13	**I**	na b	**na**	0	**R**	26	**S**
												
***Agrobacterium tumefaciens*A136**	8	**R**	8	**R**	9	**R**	9	**R**	17	**S**	10	**R**
***Agrobacterium tumefaciens*KYC6**	0	**R**	0	**R**	0	**R**	0	**R**	0	**R**	0	**R**

a EX 1: 4 mg/mL *K. africana* ethyl acetate extract; EX 2: 4 mg/mL *K. africana* dichloromethane extract; EX 3: 4 mg/mL *K. africana* methanol extract; EX 4: 4 mg/mL *K. africana* hexane extract; AMP10: ampicillin; and TE30: tetracycline.;

b na: not available.
